# Cancer Screening Test Use — United States, 2015

**DOI:** 10.15585/mmwr.mm6608a1

**Published:** 2017-03-03

**Authors:** Arica White, Trevor D. Thompson, Mary C. White, Susan A. Sabatino, Janet de Moor, Paul V. Doria-Rose, Ann M. Geiger, Lisa C. Richardson

**Affiliations:** ^1^Division of Cancer Prevention and Control, CDC; ^2^Division of Cancer Control and Population Sciences, National Cancer Institute, National Institutes of Health, Bethesda, Maryland.

*Healthy People 2020* (HP2020) includes objectives to increase screening for breast, cervical, and colorectal cancer (*1*) as recommended by the U.S. Preventive Services Task Force (USPSTF).* Progress toward meeting these objectives is monitored by measuring cancer screening test use against national targets using data from the National Health Interview Survey (NHIS) (*1*). Analysis of 2015 NHIS data indicated that screening test use remains substantially below HP2020 targets for selected cancer screening tests. Although colorectal cancer screening test use increased from 2000 to 2015, no improvements in test use were observed for breast and cervical cancer screening. Disparities exist in screening test use by race/ethnicity, socioeconomic status, and health care access indicators. Increased measures to implement evidence-based interventions and conduct targeted outreach are needed if the HP2020 targets for cancer screening are to be achieved and the disparities in screening test use are to be reduced.

NHIS is a cross-sectional household interview survey that yields data on a nationally representative sample of the civilian, noninstitutionalized population residing in the United States (*2*). Information is collected about the household, each person in the family residing in that household, and a randomly selected sample adult (aged ≥18 years) and child (if present) from each family. This analysis includes data from the cancer control supplement, sample adult questionnaire, person files, and imputed income files. For each cancer screening test, adults were asked whether they had ever received the test. Those who answered that they had received a cancer screening test were then asked when the most recent screening test occurred (*2*). For this analysis, any report of testing for cancer was considered a screening test for the purpose of estimating proportions of the population up to date with breast, cervical, and colorectal cancer screening consistent with USPSTF recommendations as of 2015 (i.e., mammography within 2 years for women aged 50–74 years; Papanicolaou [Pap] test within 3 years for women without a hysterectomy aged 21–65 or Pap test with human papillomavirus test [HPV] within 5 years for women without a hysterectomy aged 30–65 years; fecal occult blood test within 1 year, sigmoidoscopy within 5 years and fecal occult blood test within 3 years, or colonoscopy within 10 years for respondents aged 50–75 years). Crude percentages, along with corresponding 95% confidence intervals, were presented by sociodemographic and health care–access characteristics, such as source of usual care. Overall percentages were age-adjusted, with age standardized to the 2000 U.S. standard population. Because the covariate associations for colorectal cancer screening use were similar by sex, results are reported for men and women combined. Statistical testing for differences in screening test use by sociodemographic and health care–access characteristics was performed using Wald F tests. For each screening exam, screening trends over time were examined using NHIS data from 2000, 2003, 2005, 2008, 2010, 2013, and 2015. To account for changes in cervical cancer screening recommendations over time, only trends for Pap test within 3 years for women aged 21–65 years without hysterectomy were assessed. The Wald F test was used to determine whether differences in screening across the years occurred. All statistics presented are based on data weighted to account for the complex survey design of NHIS.

The final sample adult response rate was 55.2% (*2*). Mammography use remained stable from 2000 to 2015 (Figure). In 2015, 71.5% of women aged 50–74 years reported having had a mammogram within the past 2 years, which is less than the HP2020 target of 81.1% (Figure) (Table 1). Compared with other racial/ethnic groups, mammography use was lowest among American Indians/Alaska Natives (AI/AN) (56.7%). Filipino women were the only group that met the HP2020 target. Use was lower among women who were foreign-born and in the United States for <10 years (53.7%) than among those who were U.S.-born (72.1%). The proportion of women who had a mammogram increased with increasing education and income levels. Mammography use was lowest for women who reported being uninsured (35.3%) and without a usual source of health care (32.9%) (Table 1).

**FIGURE:**
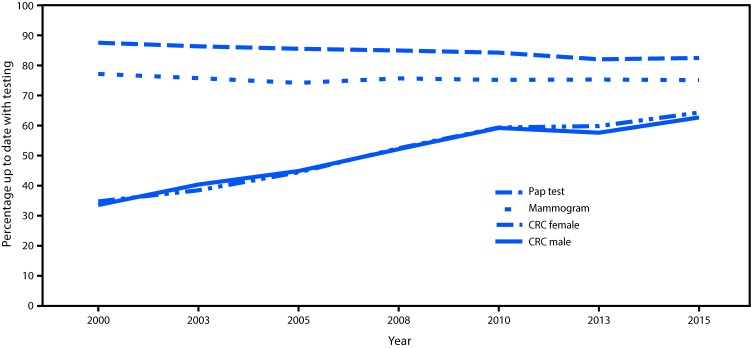
Percentage of adults who were up to date with screening for breast,* cervical,^†^ and colorectal^§^ cancers, by test, sex, and year — United States, 2000–2015. **Abbreviation:** CRC = colorectal cancer. * The U.S. Preventive Services Task Force (USPSTF) recommends mammography within 2 years for women aged 50–74 years. ^†^ USPSTF recommends Papanicolaou (Pap) test within 3 years for women aged 21–65 years without hysterectomy, or Pap test with human papillomavirus test within 5 years for women aged 30–65 years without hysterectomy. To account for changing screening recommendations over time for cervical cancer for women aged 21–65 years without hysterectomy, only trends for Pap test within 3 years for women aged 21–65 years without hysterectomy were assessed; Pap test data for 2003 are missing. ^§^ The USPSTF recommends three options for CRC screening: 1) fecal occult blood test within 1 year; 2) sigmoidoscopy within 5 years and fecal occult blood test within3 years; or 3) colonoscopy within 10 years for respondents aged 50–75 years.

**TABLE 1:** Percentage of women who received recent breast and cervical cancer screenings, by selected sociodemographic characteristics and health care access — National Health Interview Survey, United States, 2015

Characteristic	Breast cancer			Cervical cancer		
	Mammogram within ≤2 yrs*			Pap test within ≤3 yrs or Pap + HPV within ≤5 yrs^†^		
	No. (%^§^)	95% CI	p-value	No. (%^§^)	95% CI	p-value
**Overall**						
Crude	6,747 (71.6)	70.1–73.0	NA	10,477 (82.8)	81.8–83.8	NA
Age-adjusted^¶^	6,747 (71.5)	70.1–73.0		10,477 (83.0)	82.0–84.0	
Race**						
White	5,298 (71.8)	70.1–73.4	p = 0.035	7,844 (83.2)	82.0–84.3	p<0.001
Black	1,015 (74.3)	70.3–78.0		1,664 (85.3)	82.9–87.3	
American Indian/Alaska Native	86 (56.7)	43.0–69.4		171 (76.9)	66.9–84.6	
Asian	311 (66.1)	59.1–72.4		690 (75.8)	71.4–79.7	
Chinese	55 (72.3)	55.4–84.6		151 (72.0)	63.8–79.0	
Filipino	88 (81.5)	67.5–90.4		169 (88.9)	81.4–93.7	
Other Asian	168 (57.4)	48.0–66.3		370 (71.6)	65.5–77.0	
**Ethnicity^††^**						
Non-Hispanic	5,906 (71.5)	69.9–73.1	p = 0.791	8,375 (83.7)	82.6–84.8	p<0.001
Hispanic	841 (72.1)	67.8–76.0		2,102 (78.6)	76.2–80.8	
Puerto Rican	118 (78.1)	66.5–86.5		222 (79.5)	70.1–86.6	
Mexican	272 (66.2)	59.3–72.5		864 (77.0)	73.0–80.6	
Mexican-American	163 (77.2)	67.4–84.8		417 (79.0)	72.8–84.1	
Central/South American	144 (74.6)	64.6–82.6		359 (80.6)	74.5–85.5	
Other Hispanic	118 (78.1)	66.5–86.5		240 (80.5)	72.1–86.8	
**Age group (yrs)**						
21–30	—^§§^	—^§§^	p = 0.556	2,594 (78.3)	75.9–80.5	p<0.001
31–40	—^§§^	—^§§^		2,647 (87.2)	85.4–88.9	
41–50	—^§§^	—^§§^		2,180 (84.6)	82.5–86.5	
51–65	—^§§^	—^§§^		3,056 (82.0)	80.2–83.7	
50–64	4,312 (71.3)	69.4–73.1		—^§§^	—^§§^	
65–74	2,435 (72.2)	69.7–74.5		—^§§^	—^§§^	
**Sexual orientation**						
Gay	94 (77.2)	65.0–86.1	p = 0.380	177 (74.6)	64.9–82.4	p = 0.006
Straight	6,509 (71.8)	70.3–73.2		10,000 (83.3)	82.2–84.2	
Bisexual	26 (38.3)^¶¶^	14.5–69.5^¶¶^		161 (77.9)	68.5–85.1	
**Period of U.S. residence**						
U.S.-born	5,692 (72.1)	70.5–73.6	p = 0.034	8,232 (84.5)	83.3–85.5	p<0.001
In U.S. <10 yrs	74 (53.7)	40.2–66.8		467 (67.3)	62.2–72.0	
In U.S. ≥10 yrs	971 (70.0)	65.9–73.8		1,760 (79.3)	76.7–81.6	
**Education**						
Less than high school	867 (60.3)	55.7–64.7	p<0.001	1,215 (71.2)	67.6–74.5	p<0.001
High school graduate/GED	1,698 (68.3)	65.3–71.2		2,130 (76.4)	73.8–78.9	
Some college/Associate degree	2,187 (71.0)	68.2–73.8		3,436 (83.1)	81.1–84.9	
College graduate	1,970 (78.9)	76.4–81.2		3,670 (89.5)	88.1–90.7	
**Percentage of federal poverty threshold**						
<139	1,571 (58.7)	55.0–62.3	p<0.001	2,960 (75.2)	72.9–77.4	p<0.001
139–250	1,323 (63.4)	59.3–67.4		2,075 (78.2)	75.5–80.7	
251–400	1,311 (73.8)	70.5–76.9		1,960 (82.3)	79.9–84.4	
>400	2,542 (78.8)	76.6–80.9		3,481 (89.7)	88.2–90.9	
**Usual source of health care**						
None or hospital emergency department	393 (32.9)	26.9–39.6	p<0.001	1,406 (65.1)	61.5–68.6	p<0.001
Has usual source	6,352 (73.8)	72.3–75.3		9,069 (85.5)	84.5–86.5	
**Health care coverage**						
Private	4,186 (76.7)	74.9–78.5	p<0.001	6,739 (86.8)	85.7–87.8	p<0.001
Military	222 (74.5)	66.1–81.3		263 (92.9)	88.2–95.8	
Public only	1,951 (64.3)	61.4–67.1		2,118 (78.4)	75.9–80.7	
Uninsured	370 (35.3)	29.2–41.9		1,318 (63.8)	60.3–67.2	

**Abbreviations:** CI = confidence interval; GED = General Educational Development certificate; HPV = human papillomavirus; NA = not applicable; Pap = Papanicolaou.

* Among women aged 50–74 years.

^†^ Pap test for women without hysterectomy either within 3 years for women aged 21–65, or Pap with HPV test within 5 years for women aged 30–65 years.

^§^ Weighted percentages. Overall percentages presented as crude and age-adjusted estimates; other percentages are crude estimates.

^¶^ Age-standardized to the 2000 U.S. standard population.

** p-value testing for differences across four primary race groups.

^††^ p-value testing for differences between Hispanic and non-Hispanics.

^§§^ Not estimated for these age groups.

^¶¶^ Relative standard error >30%.

From 2000 to 2015, the overall trend for cervical cancer screening (Pap test) use declined (Figure). In 2015, 83% of women reported being up to date with cervical cancer screening, which is below the HP2020 target of 93.0% (Figure) (Table 1). Cervical cancer screening use was lowest among Asian women (75.8%), especially Chinese (72.0%) and other Asian women (71.6%). Hispanics (78.6%) reported lower screening than did non-Hispanics (83.7%). Compared with all other age groups, women aged 21–30 years reported the lowest cervical cancer screening test use (78.3%). Women who were foreign-born, regardless of their duration of U.S. residence, had lower screening test use than U.S.-born women. The proportion of women reporting cervical cancer screening use increased with education and income levels. Cervical cancer screening use was lower among women without a usual source of health care (65.1%) than among women who had a usual source of care (85.5%). Compared with women who had insurance coverage, cervical cancer screening test use was lowest (63.8%) among uninsured women (Table 1).

From 2000 to 2015, colorectal cancer test use increased, but did not reach the HP2020 target of 70.5% (Figure). During 2015, 62.4% of men and women reported colorectal cancer screening test use consistent with USPSTF recommendations. By racial group, colorectal cancer screening use was lowest among AI/ANs (48.4%) (Table 2). By ethnicity, Hispanics reported lower screening test use (47.4%) than did non-Hispanics (64.2%). Reported screening was lower among persons aged 50–64 years (57.9%) than among persons aged 65–75 years (71.8%). Foreign-born persons reported lower use of colorectal cancer screening (52.3% [U.S. residence ≥10 years], 36.3% [U.S. residence <10 years]) than did U.S.-born persons (64.6%). As education and income levels increased, the proportion of persons who received colorectal cancer screening increased. Lowest colorectal cancer screening use was reported by persons without a usual source of health care (26.3%) and persons who were uninsured (25.1%).

**TABLE 2:** Percentage of adults who received recent colorectal cancer screenings,* by selected sociodemographic characteristics and health care access — National Health Interview Survey, United States, 2015

Characteristic	No. (%^†^)	95% CI
**Overall**		
Crude	12,650 (62.4)	61.1–63.7
Age–adjusted^§^	12,650 (62.4)	61.1–63.8
Race^¶,^**		
White	10,051 (63.7)	62.2–65.2
Black	1,777 (59.3)	56.0–62.5
American Indian/Alaska Native	160 (48.4)	38.3–58.7
Asian	595 (52.1)	46.7–57.4
Chinese	111 (56.0)	44.5–67.0
Filipino	161 (54.7)	43.2–65.7
Other Asian	323 (49.7)	43.4–56.0
**Ethnicity^¶,††^**		
Non-Hispanic	11,163 (64.2)	62.7–65.6
Hispanic	1,487 (47.4)	44.1–50.8
Puerto Rican	192 (63.2)	54.3–71.2
Mexican	501 (36.0)	31.0–41.4
Mexican-American	307 (49.8)	41.9–57.8
Central/South American	240 (52.6)	43.2–61.8
Other Hispanic	247 (51.6)	43.8–59.4
**Age group (yrs)^¶^**		
50–64	7,947 (57.9)	56.2–59.6
65–75	4,703 (71.8)	70.0–73.6
**Sexual Orientation^§§^**		
Gay	210 (69.3)	60.6–76.8
Straight	12,195 (62.5)	61.1–63.8
Bisexual	49 (59.3)	36.6–78.6
**Period of U.S. residence^¶^**		
U.S.-born	10,716 (64.6)	63.1–66.0
In U.S. <10 yrs	133 (36.3)	26.6–47.3
In U.S. ≥10 yrs	1,781 (52.3)	49.3–55.2
**Education^¶^**		
Less than high school	1,681 (46.7)	43.5–50.0
High school graduate/GED	3,275 (58.2)	55.9–60.6
Some college/Associate degree	3,896 (63.5)	61.2–65.6
College graduate	3,754 (70.7)	68.7–72.7
**Percentage of federal poverty threshold^¶^**		
<139	2,702 (46.9)	44.4–49.5
139–250	2,432 (56.1)	52.9–59.1
251–400	2,455 (62.6)	59.6–65.5
>400	5,060 (70.0)	68.2–71.8
**Usual source of health care^¶^**		
None or hospital emergency department	997 (26.3)	22.5–30.4
Has usual source	11,651 (65.2)	63.8–66.6
**Health care coverage^¶^**		
Private	7,628 (65.6)	63.9–67.2
Military	702 (77.6)	72.8–81.7
Public only	3,494 (60.1)	57.9–62.2
Uninsured	790 (25.1)	20.9–29.9

**Abbreviations:** CI = confidence interval; GED = General Educational Development certificate.

* Includes fecal occult blood test within 1 year, sigmoidoscopy within 5 years and fecal occult blood test within 3 years, or colonoscopy within 10 years for persons aged 50–75 years.

^†^ Weighted percentages. Overall percentages presented as crude and age–adjusted estimates; other percentages are crude estimates.

^§^ Age-standardized to the 2000 U.S. standard population.

^¶^ p<0.001.

** p-value testing for differences across four primary race groups.

^††^ p-value testing for differences between Hispanic and non-Hispanics.

^§§^ p = 0.038.

## Discussion

Cancer screening in the United States remains below HP2020 targets. A previous study of cancer screening using data from the 2013 NHIS found that overall use of screening tests was below HP2020 targets, with no improvements from 2010 to 2013 for breast, cervical, or colorectal cancer (*3*). Based on these more recent data, the overall trend from 2000 to 2015 demonstrates that colorectal cancer screening increased, breast cancer screening was stable, and cervical cancer screening declined slightly. Few subgroups met HP2020 targets in 2015, with many groups remaining far below targets, and disparities in use of cancer screening tests exist based on race, ethnicity, income, and education.

The progress in increasing use of colorectal cancer screening is promising, but more needs to be done if the HP2020 target is to be achieved. The lack of progress for breast and cervical cancer screening use highlights the need for more initiatives to reach persons facing barriers to screening. Persons without a usual source of health care and the uninsured had the lowest test use, with the overwhelming majority of the uninsured not up to date with breast and colorectal cancer screening. The Affordable Care Act has helped to reduce such barriers by expanding insurance coverage and eliminating cost sharing, in most insurance plans, for preventive services such as breast, cervical, and colorectal cancer screening rated A and B by the USPSTF.^†^ Further, CDC’s Colorectal Cancer Control Program helps states and tribes increase colorectal cancer screening use by reducing some barriers and promoting the use of evidence-based interventions to increase screening (*4*). The National Breast and Cervical Cancer Early Detection Program^§^ provides free or low-cost screening to medically underserved women.

Mammography use among AI/AN declined from 73.4% in 2013 (*3*) to 56.7% 2015. From 1990 to 2009, breast cancer death rates declined for white women, but increased slightly among AI/AN women (*5*). Reasons for this decline are unclear and warrant further investigation. However, data from this analysis indicate that factors associated with lower mammography use include poverty and lack of insurance coverage or a usual source of health care. In addition, because of the small sample size and unstable estimates for AI/AN women, error cannot be ruled out as a potential explanation for this pattern. Lower mammography use might lead to breast cancer diagnosis at later stages and contribute to racial disparities in mortality. The National Breast and Cervical Cancer Early Detection Program supports 11 AI/AN tribes and tribal organizations to increase screening use in these communities (*4*,*6*).

The findings in this report are subject to at least five limitations. First, the screening questions did not distinguish whether the test was performed for screening or diagnostic purposes; however, a person might be considered effectively screened in either instance. Second, data were self-reported and were not verified by medical records. Third, the overall response rate was 55.2%, and nonresponse bias is possible, despite adjustments for nonresponse. Fourth, sample sizes were small and not age-adjusted for some subgroups. Comparisons of subgroup rates to national targets should be interpreted with caution because targets were based on improvement from the 2008 baseline values for the national age-adjusted rate. In addition, consideration should be given to the fact that targets were designed to be met by 2020, not 2015. Finally, screening recommendations and questions have changed over time. In 2012, screening every 5 years with Pap and HPV tests was added as an option for women aged 30–65 years. It is unclear whether this change might have extended screening intervals for women and thus contributed to the slight decline in cervical cancer screening. Attempts were made to account methodologically for changes in recommendations and questions by using consistent definitions across years. Because hysterectomy status was unknown for 2003, Pap test data for that year were excluded Screening measures for the trend analysis were defined according to the 2000 method, which makes assumptions for cases with only partial timing data (i.e. respondent did not provide enough timing detail to determine if the test came within the recommended time interval). This source of bias results in slightly higher estimates but allows for fair comparisons over time. Accordingly, percentages for 2015 in the trend analysis differ slightly from those reported in the tables.

These findings might inform future activities to increase the use of screening tests as recommended. Some progress has been achieved toward meeting the HP2020 objective for colorectal cancer screening, but the trend for mammography use has remained static, and cervical cancer screening is declining. Substantial disparities persist for some subgroups, including persons without health insurance or a usual source of health care. The National Breast and Cervical Cancer Early Detection Program can provide access to timely breast and cervical cancer screening and diagnostic services for low-income, uninsured, and medically underserved women. For persons with access to health care, evidence-based interventions, such as provider and patient reminders about screening, can increase cancer screening rates (*7*). Innovative approaches are needed to reach some racial and ethnic minorities and medically underserved populations to improve the use of cancer screening test use toward the HP2020 targets.

**Summary**

**What is already known about this topic?**

Screening can lead to early detection of breast, cervical and colorectal cancer, when cancers might respond better to treatment, thereby reducing deaths. *Healthy People 2020* (HP2020) set targets for screening based on recommendations from the U.S. Preventive Services Task Force. Screening disparities exist for some groups defined by sociodemographics and access to health care.

**What is added by this report?**

Since 2013, some progress toward meeting the HP2020 objective for colorectal cancer screening has occurred, but the trend for breast cancer screening has been static, and cervical cancer screening is declining. Disparities in screening persisted by race, ethnicity, education, and income. The uninsured and persons without a usual source of care had screening use far below the HP2020 targets.

**What are the implications for public health practice?**

Progress toward achieving the HP2020 targets will require implementation of evidence-based interventions to increase cancer screening. Such interventions can be both provider- and patient-oriented. Screening among some racial and ethnic minorities and medically underserved populations is suboptimal and innovative approaches to eliminate these disparities might be needed.

## Footnotes

* https://www.uspreventiveservicestaskforce.org/Page/Name/recommendations.

† U.S. Preventive Services Task Force (USPSTF) A and B grades are defined as follows: A, USPSTF recommends the service and there is high certainty that the net benefit is substantial; B, USPSTF recommends the service and there is high certainty that the net benefit is moderate, or there is moderate certainty that the net benefit is moderate to substantial. https://www.uspreventiveservicestaskforce.org/Page/Name/grade-definitions.

§ https://www.cdc.gov/cancer/nbccedp.
